# Morpho-Colorimetric Characterization of the Sardinian Endemic Taxa of the Genus *Anchusa* L. by Seed Image Analysis

**DOI:** 10.3390/plants9101321

**Published:** 2020-10-06

**Authors:** Emmanuele Farris, Martino Orrù, Mariano Ucchesu, Arianna Amadori, Marco Porceddu, Gianluigi Bacchetta

**Affiliations:** 1 Dipartimento di Chimica e Farmacia, Università di Sassari, Via Piandanna 4, 07100 Sassari, Italy; emfa@uniss.it (E.F.); 30049374@studenti.uniss.it (A.A.); 2 Independent researcher, via Nazionale, 09023 Monastir (CA), Italy; martino.orru@gmail.com; 3 Centre for the Conservation of Biodiversity (CCB), Life and Environmental Sciences Department, University of Cagliari (DiSVA), Viale S. Ignazio da Laconi 11-13, 09123 Cagliari, Italy; marianoucchesu@unica.it (M.U.); bacchet@unica.it (G.B.); 4 Sardinian Germplasm Bank (BG-SAR), Hortus Botanicus Karalitanus (HBK), University of Cagliari, Viale S. Ignazio da Laconi, 9-11, 09123 Cagliari, Italy

**Keywords:** Alkanets, Boraginaceae, endemic species, Mediterranean vascular flora, Tyrrhenian islands

## Abstract

In this work, the seed morpho-colorimetric differentiation of the Sardinian endemic species of *Anchusa* (Boraginaceae) was evaluated. In Sardinia, the *Anchusa* genus includes the following seven taxa: *A. capellii*, *A. crispa* ssp. *crispa*, *A. crispa* ssp. *maritima*, *A. formosa*, *A. littorea*, *A. montelinasana*, and *A. sardoa*. Seed images were acquired using a flatbed scanner and analyzed using the free software package ImageJ. A total of 74 seed morpho-colorimetric features of 2692 seed lots of seven taxa of *Anchusa* belonging to 17 populations were extrapolated and used to build a database of seed size, shape, and color features. The data were statistically elaborated by the stepwise linear discriminant analysis (LDA) to compare and discriminate each accession and taxon. In addition, the seed morpho-colorimetric differences among coastal and mountainous taxa were evaluated. Considering the ecological conditions, the LDA was able to discriminate among the *Anchusa* taxa with a correct identification of 87.4% and 90.8% of specimens for mountainous and coastal plants, respectively. Moreover, the LDA of the 17 populations of *Anchusa* showed a low separation among species and populations within the coastal group, highlighting how the long-distance dispersal by flotation on the sea water surface and the pollination network may influence the similarity patterns observed. In addition, a misattribution was observed for *A. crispa* ssp. *crispa*, which was misclassified as *A. crispa* ssp. *maritima* in 14.1% of cases, while *A. crispa* ssp. *maritima* was misidentified as *A. crispa* ssp. *crispa* in 21.1% of cases, highlighting a close phenotypic relationship between these two taxa. The statistical results obtained through the seed image analysis showed that the morpho-colorimetric features of the seeds provide important information about the adaptation and evolution of *Anchusa* taxa in Sardinia.

## 1. Introduction

In the Old World, the Boragineae tribe (Boraginaceae family) consists of 16 genera and about 170 taxa. The distribution range is centered in the Mediterranean Basin and in the Middle East, but it is present throughout Europe and tropical Africa, with a second minor center in the Cape region [1,2,3,4,5]. Currently, about 40 taxa [3,6] of the genus *Anchusa* L. are known in the Mediterranean Basin, with a main diversity center in the southern part of the Balkan peninsula [7].

In terms of floristic richness and number of endemism, the islands of Corsica and Sardinia are two meso-scale hotspots in the macro-scale hotspot of the Tyrrhenian islands and the mega-hotspot of the Mediterranean Basin [8,9,10]. At the same time, the two islands represent an important center of differentiation of the genus *Anchusa* due to the presence of eight endemic taxa [6,11]. These endemic taxa have a narrow or even dot-like distribution range and grow in different habitats, from coastal to mountain regions [6]. Bigazzi and Selvi [3] hypothesized that these species have a common genetic pool of tertiary origin. The Oligo-Miocenic rotation of the Corso-Sardinian complex and the subsequent geological events could have led to the fragmentation and isolation of some *Anchusa* populations that would have undergone genetic drift and/or a schizogenetic differentiation. The genus *Anchusa* is considered to be recent in the ambit of the family Boraginaceae, dating to the Pliocene–Pleistocene [12], and Sardinian-Corsican taxa are considered to be involved in a recent, still ongoing process of differentiation and speciation [6,13]. The very restricted range and low population size of the *Anchusa* taxa present, in Corsica and Sardinia, give the endemism a very precarious conservation status [6]. In Sardinia, the genus *Anchusa* consists of about 13,000 individuals growing in different populations and belonging to seven allopatric endemic taxa, four of which (*A. littorea* Moris (350 individuals), *A. sardoa* (Illario) Selvi & Bigazzi (1500), *A. crispa* Viv. ssp. *crispa* (2100), and *A. crispa* Viv. ssp. *maritima* (Vals.) Selvi & Bigazzi (6000)) occur in coastal habitats, while the other three (*A. capellii* Moris (1000), *A. formosa* Selvi, Bigazzi & Bacch. (2150), and *A. montelinasana* Angius, Pontec. & Selvi (200)) are typical of mountain habitats [13,14]. The mountain populations are well preserved as compared with the coastal ones, which are in a precarious state of conservation due to high anthropogenic pressure. In particular, the negative impact of human activities related to mass tourism (trampling, building on the dunes, and introduction of alien plants) has been observed on several populations of *A. crispa* on the west coast of Corsica and on *A. crispa* and *A. littorea* in Sardinia [13,15]. Moreover, *A. sardoa* is also strongly threatened due to excessive trampling by tourists [16,17,18].

The seed is the most important plant reproductive unit responsible for the evolutionary success of Spermatophytes. It is an important means of dispersion, but it also gives a large amount of information about species’ adaptation and evolution [19]. During the last few years, image analyses on seeds and fruits has provided increasing support to botanical studies by enabling taxonomic discrimination of plant species on the basis of their morpho-colorimetric characteristics. In particular, it has been successfully applied for *Astragalus maritimus* and *A. verrucosus* [20], *Astragalus* sect. *Melanocercis* [21] *Lavatera triloba* aggregate [22], *Astragalus tragacantha* complex [23] and *Lavatera* L., *Malva* L., and *Cistus* L. [24,25]. Linear discriminant analysis (LDA) models have proven to be effective for the discrimination and quality classification of seeds when combined with imaging techniques [26,27]. Morpho-colorimetric studies have also resulted in the taxonomic differentiation of *Juniperus* L. [28], *Medicago* L. sect. *Dendrotelis* [29], and the *Paeonia mascula* group [26]. Moreover, the validity of the image analysis technique has successfully been applied to discriminate between cultivated plants such as *Vitis vinifera* ssp. *vinifera* [30,31], *Olea europaea* [32], *Malus domestica* [33,34], *Prunus domestica* [35,36], and *Cucumis melo* [37].

On the basis of a previous study carried out by Bacchetta et al. [13], in which they investigated the phylogenetic relationships of the endemic taxa of the genus *Anchusa* present in Sardinia, this work aims to characterize these taxa on the basis of seed morpho-colorimetric features through image analysis techniques. In particular, the main goals of this work were to investigate the relationships between the *Anchusa* species and to evaluate if similarities are present among the seeds, considering the ecological conditions of the sites where the different taxa grow.

## 2. Results

Principal component analysis (PCA) was performed on the seed morpho-colorimetric data. The first 20 main components explained 99% of the original variation between morpho-colorimetric parameters (see Table S1). Accordingly, the first 20 principal components (PCs) were used for LDA. According to the results obtained from the PCA, and focusing on the first two main components, PC1 and PC2 explained 31.2% and 19.2% of the original variation, respectively (Figure S1). The top discriminant features for PC1 were, principally, colorimetric (e.g., GreenIntDen, GrIntDen, GrAverage, GrMedian, GrMedian, and RedIntDen (Figure S2)), while for PC2, they were principally morphometric (e.g., CHull, Perim, CArea, EquivEllAr, ArBBox, ArEquivD, Pixels, PerEquivD, and Area (Figure S3)).

**Figure 1:**
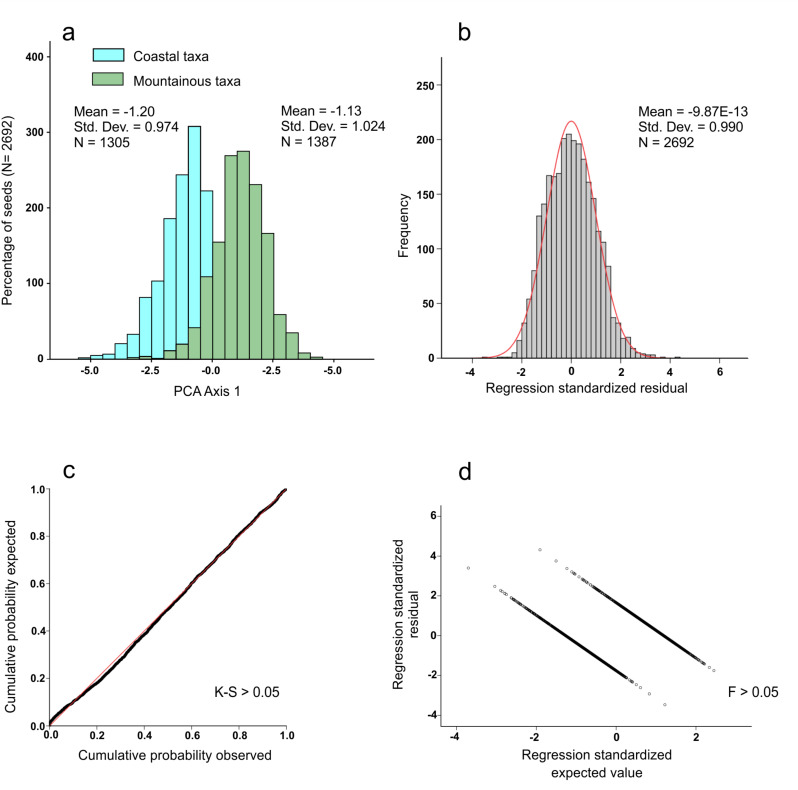
(**a**) Graphical representation of the discriminating function scores for coastal and mountainous *Anchusa* taxa; (**b**) Histogram of the standardized residuals; (**c**) Dispersion plot of the standardized residuals tested with Levene’s test (F); (**d**) Normal probability plot (P-P) tested with Kolmogorov–Smirnov’s test (K-S).

First, discrimination analysis was performed considering the environmental conditions of the *Anchusa* taxa, which allowed us to divide them into two ecological groups, i.e., coastal (*A. crispa* ssp. *crispa*, *A. crispa* ssp. *maritima*, *A. littorea*, and *A. sardoa*) and mountainous (*A. capellii*, *A. formosa*, and *A. montelinasana*) taxa. In this case, an overall percentage of correct identification of 89.0% was reached, ranging from 87.4% for the mountainous taxa to 90.8% for the coastal taxa (Table 1 and Figure 1). The most discriminant features were related to seven colorimetric (PC1) and nine morphometric variables (PC2) (Supplementary Materials).

**Figure 2:**
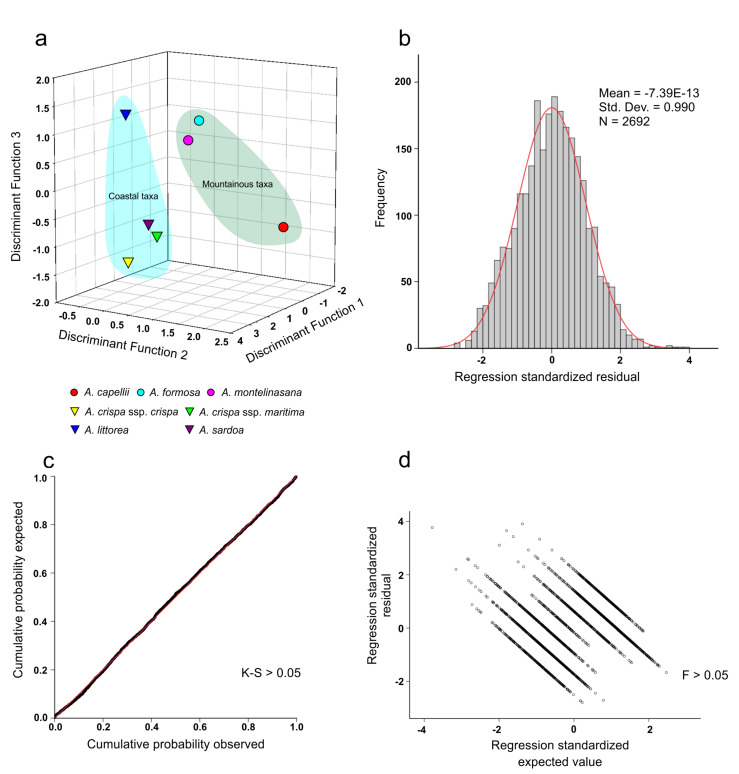
Distribution of the taxonomical groups’ centroids on the basis of three available discriminant functions. (**a**) Graphical representation of the discriminant scores of the seven *Anchusa* taxa; (**b**) Histogram of the standardized residuals; (**c**) Dispersion plot of the standardized residuals tested with Levene’s test (F); (**d**) Normal probability plot (P-P) tested with Kolmogorov–Smirnov’s test (K-S).

In order to identify each taxon, all *Anchusa* taxa were individually compared with each other. The second discrimination analysis provided an overall percentage of correct classification of 59.3%, with a range from 34.9% to 82.9% (Table 2 and Figure 2). High discrimination performance was obtained for *A. sardoa* (82.9%) and *A. littorea* (81.2%) (Table 2). Misattributions were observed between *A. montelinasana* and *A. formosa* in 26.0% of cases (Table 2). Moreover, a misattribution was observed between *A. crispa* ssp. *crispa* and *A. crispa* ssp. *maritima*. In particular, *A. crispa* ssp. *crispa* was misidentified as *A. crispa* ssp. *maritima* in 14.1% of cases and *A. crispa* ssp. *maritima* was misidentified as *A. crispa* ssp. *crispa* in 21.1% of cases (Table 2).

**Figure 3:**
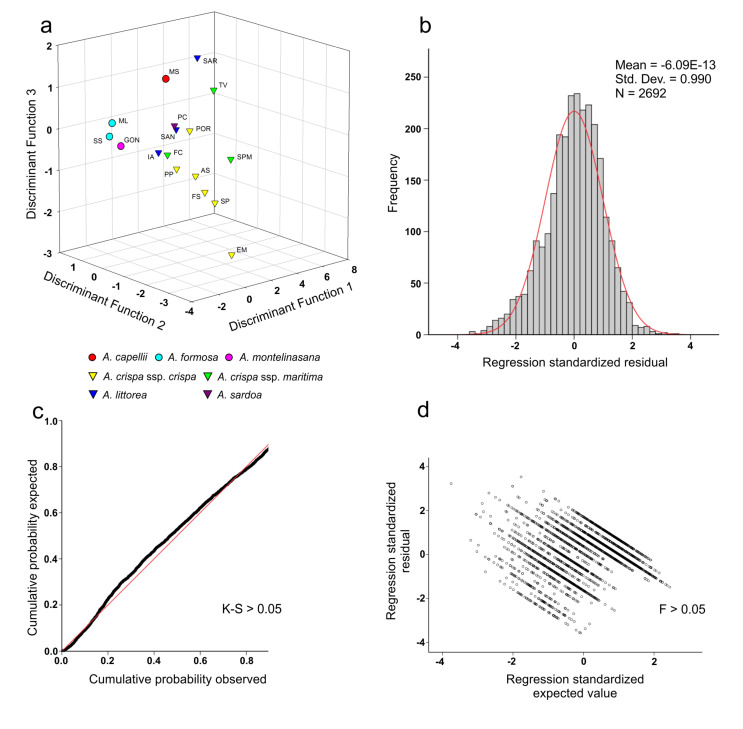
Distribution of the taxonomical groups’ centroids on the basis of three available discriminant functions. (**a**) Graphical representation of the discriminant scores of the 17 *Anchusa* populations; (**b**) Histogram of the standardized residuals; (**c**) Dispersion plot of the standardized residuals tested with Levene’s test (F); (**d**) Normal probability plot (P-P) tested with Kolmogorov–Smirnov’s test (K-S).

Furthermore, the 17 *Anchusa* accessions were compared at the population level (Figure 3). An overall percentage of correct identification of 47.0% was reached (Table S2). In this case, a correct classification range between 24.6% (EM) and 74.8% (PC) was recorded (Table S2). Population MS of *A. capellii* was misattributed to population TV of *A*. *crispa* ssp. *maritima* in 12.9% of cases (Table S2), while population SP of *A. crispa* ssp. *crispa* was misidentified as the EM population of *A. crispa* ssp. *maritima* in 19.3% of cases (Table S2). In addition, the AS population of *A. crispa* ssp. *maritima* was misattributed to population POR of *A. crispa* ssp. *maritima* in 16.4% of cases (Table S2). Regarding the two populations of *A. formosa*, the results of the LDA showed a misclassification between them in 20.5% of cases (Table S2). Misattribution was also highlighted among the GON population of *A. montelinasana* and the ML and SS populations of *A. formosa* in 18.4% and 21.6% of the cases, respectively (Figure 3 and Table S2). 

## 3. Discussion

It was previously shown that the mountain taxa of the genus *Anchusa* in Sardinia are older (three species with dot-like distribution). Accordingly, the mountain taxa are considered to be the ancestors of the coastal taxa [6,13]. The results, presented here, confirm the clear separation among the taxa of the mountain and coastal groups. The populations of taxa included in each of the two main ecological groups analyzed in this work (i.e., mountain and coastal) share very similar ecological conditions, whereas the two groups are differentiated by very different abiotic conditions of the sites where populations grow. In detail, the taxa from mountain populations grow at sites with acidic soils and meso- to supra-Mediterranean humid bioclimate, while coastal populations are found on coastal sands characterized by thermo-Mediterranean dry bioclimate. As detected in this study, the correct classification percentages among coastal and mountainous *Anchusa* taxa endemic to Sardinia are higher than 90%. These data confirm the average separation between the coastal and mountain groups of taxa. Therefore, we may suppose that these two main groups highlighted by our analyses are strongly related to similar ecological conditions (of populations within groups). In addition, the low separation among taxa and populations within the coastal group confirms what has already been highlighted by both morphological [3,5] and genetic analyses [6,13]. 

When we move to the comparison among different taxa, the percentages of correct identification of the Sardinian *Anchusa* taxa are lower than the correct classification percentages among coastal and mountainous *Anchusa* groups, ranging between 34.9% and 82.9% and averaging 56.5%. In this case, only four taxa (*A. crispa* ssp. *crispa*, *A. littorea*, *A. sardoa*, and *A. capellii*) achieved a correct classification percentage that exceeded 70%. The results showed how *A. crispa* ssp. *maritima* obtained the lowest classification among the seven analyzed taxa; it was misattributed to *A. crispa* ssp. *crispa* and *A. sardoa*. Selvi and Bigazzi [4] already suspected the subspecific status of the eastern populations of *A. crispa*, and our data confirmed that no clear separation existed among *A. crispa* ssp. *maritima* and the other two taxa of *Anchusa* from northwestern Sardinia (*A. crispa* ssp. *crispa* and *A. sardoa*). Among the mountain taxa, *A. formosa* and *A. montelinasana* showed a low percentage of correct classification due to misattribution among them. In this case, the close locations of *A. formosa* and *A. montelinasana* can sustain a continuous, if weak, gene flow through pollination, which can be the basis of a common set of seed features. Furthermore, the three-dimensional (3D) graphical representation of the discriminant analysis of the seven *Anchusa* taxa shows a clear distinction between coastal and mountain taxa. Overall, at the species level, our analyses effectively reflect the different ecological and geographical conditions of each taxon. Figure 2a depicts two distinct ecological groups of taxa (coastal vs. mountain), and within each group the taxa that are more geographically isolated and ecologically differentiated are clearly highlighted. Within the coastal group, *A. littorea* is the only annual species growing in very harsh conditions in the southwestern part of the island (all the other coastal taxa grow in the north-western side, in less dry sites); within the mountain group, *A. capellii* is the only taxon growing in central mountains, in less oceanic and more temperate (submediterranean) conditions with respect to the other two mountain taxa.

At the population level, the percentage of correct identification was even lower, averaging 47.0%. The 3D graphical representation of the discriminant analysis of the 17 *Anchusa* populations analyzed on the basis of the seeds’ features highlighted a low separation among the different populations. It is noteworthy that several populations attributed to different coastal taxa are very close, particularly, the *A. crispa* ssp. *crispa* and *A. crispa* ssp. *maritima* populations and the three populations of *A. littorea*. Among mountain populations, a low separation was observed between the *A. formosa* and *A. montelinasana* populations. Moreover, the analysis showed a clear differentiation between *A. littorea* and the other taxa. The location of *A. littorea* outside the group of coastal taxa could be due to the geographic distribution of the coastal taxa and its relative isolation from the other coastal taxa that are located in northern Sardinia. This difference could also be related to the life cycle of this species. In fact, *A. littorea* is the only taxon that shows a therophytic habit and a strongly abbreviated life cycle with respect to the other biennial/perennial endemic taxa of *Anchusa* present in Sardinia [13]. In addition, our results showed a close relationship among *A. crispa* ssp. *crispa,* and *A. crispa* ssp. *maritima*, confirming the observations reported by Selvi and Bigazzi [4], who attributed this similarity to their similar ecologies and uncompleted differentiation processes.

It has been previously demonstrated that the coastal taxa studied here have a double method of seed dispersal, i.e., a short-distance dispersal mediated by ants (myrmecochory and dyszoochory) and a long-distance dispersal (LDD) by sea water transport [38]. LDD is central to species expansion following climate change, the re-colonization of disturbed areas, and the control of pests [39]. LDD by flotation on the sea water surface can influence the similarity patterns observed here, because it is probably driven more by sea current dynamics than by the linear distances among populations. Therefore, we cannot exclude the possibility of seed exchange among far coastal populations, particularly those located on the northwestern side of the island, where coastal currents are mainly from north to south [40]. Conversely, this possibility is much less likely to happen among mountain populations, because a potential long-distance dispersal of their seeds (still to be demonstrated) can be mediated only by freshwater runoff after heavy rains, and, if present, can only be unidirectional from the top of the mountains to the bottom.

Additionally, the low percentages of correct identification at the population level, for both the coastal and the mountain groups of Sardinian *Anchusa*, could also be due to uncompleted differentiation processes among closely related taxa (i.e., *A. crispa* ssp. *crispa*, *A. crispa* ssp. *maritima*, and *A. sardoa*), whose gene flow can be maintained by the pollinators network. For another Eudicot species (*Sorbus,* Rosaceae) pollinated by generalist insects, it was previously shown that c. 2% of the pollen is moved long distances (12–16 km) over fragmented landscapes, assuring functional connections among fragmented subpopulations by gene flow through pollination [41].

The discriminant analysis applied to the different Sardinian endemic *Anchusa* taxa through the analysis of the morpho-colorimetric characteristics of the seeds has highlighted how the ecological characteristics in which these taxa grow are important factors that can generate diversity and similarity even within populations of the same taxon. The morpho-colorimetric database of the Sardinian endemic *Anchusa* taxa that was built with this study will be a valuable resource for future studies. The database can be increased with new accessions of the *Anchusa* species in order to study the diversity existing between the endemic taxa present in Sardinia and others that grow in the Mediterranean territories. 

## 4. Materials and Methods 

### 4.1. Diagnostic Characteristics of Mericarps of the Studied Taxa

In this study, seed morpho-colorimetric parameters of the Sardinian endemic taxa of the genus Anchusa were analyzed. The fruit of these taxa is constituted by mericarpids. In A. formosa, the mericarpids are obliquely ovoid, small, c. 2 × 1.5 mm, with a weak basal rim and a blackish, minutely papillose surface, with a reticulation of blunt ridges. A. montelinasana is morphologically close to A. capellii, but differs from the latter due to a combination of quantitative and qualitative characteristics of taxonomic value. The mericarpids of A. montelinasana are transversely ovoid, c. 2.1 × 1.7 mm, blackish, and have a finely tuberculate coat surface, with a reticulation of blunt ridges and a thin basal annulus, while A. capellii mericarpids are c. 2.8 × 1.8 mm and obliquely erect, with a distinct basal annulus, a sparsely tuberculate surface, and dark brown coloration. The mericarpids of A. sardoa are obliquely ovoid, c. 2.5 × 1.5 mm, with a pointed apex and a surface that is light brown-greyish and densely tuberculate, with a sparse reticulation of blunt ridges. A. littorea mericarps are light grey-brown, small, and 1.5–2 × 0.5–1 mm, with a lateral beak, a thin basal annulus, and a finely tuberculate surface. The mericarpids of A. crispa are obliquely ovoid and c. 2.1 × 1.3 mm, with a blunt apex and a greyish, tuberculated surface, with a reticulation of blunt ridges [13]. The difference between A. crispa ssp. crispa and A. crispa ssp. maritima is related to genetic data [6].

### 4.2. Plant Material

**Figure 4:**
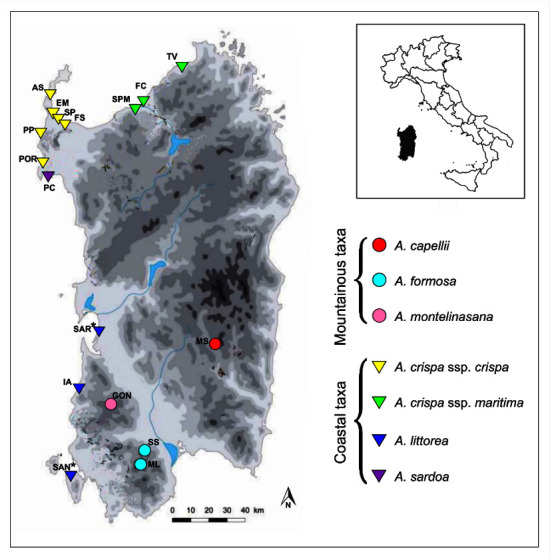
Populations of the *Anchusa* taxa analyzed in this study. * Extinct populations.

The mericarpids (hereafter seeds) of seven taxa of *Anchusa* belonging to 15 existing and two extinct Sardinian populations, with a total of 34 accessions, were analyzed (Figure 4 and Table S3). The accessions, collected over a period of 14 years, were manually cleaned and stored at −25 °C at the Sardinian Germplasm Bank (BG-SAR) of Hortus Botanicus Karalitanus of the University of Cagliari on the basis of established international protocols [42,43,44]. Each accession reported in Table S3 corresponds to a sample of seeds collected in a single year and at the time of natural dispersal from a unique population, which was stored separately by ensuring that there was no mixing of the seeds from other accessions. Seed lots of two extinct populations of *A. littorea* coming from herbarium specimens preserved at the Herbarium CAG (University of Cagliari, Italy) were added to the dataset.

### 4.3. Seed Image Analysis

**Figure 5:**
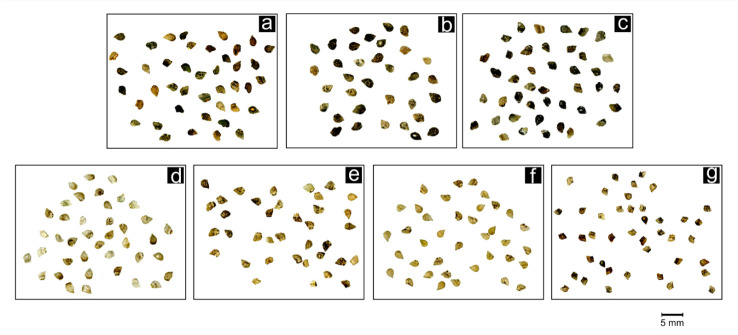
Examples of the seed image scans of the taxa analyzed. (**a**) *A. capellii*; (**b**) *A. formosa*; (**c**) *A. montelinasana*; (**d**) *A. crispa* ssp. *crispa*; (**e**) *A. crispa* ssp. *maritima*; (**f**) *A. littorea*; (**g**) *A. sardoa*.

Images (Figure 5) were acquired from a total of 2692 *Anchusa* seeds using a flatbed scanner (Epson Perfection V550) with a digital resolution of 800 dpi for a scanning area not exceeding 1024 × 1024 pixels [45].

Digital seed images were analyzed using the free software package ImageJ v. 1.49 [46]. A plugin, Particles8 [47] was used to measure the seed morpho-colorimetric features (Table 3). A total of 74 seed morpho-colorimetric parameters of the seed lots related to Sardinian endemic taxa of the *Anchusa* genus were used to build a database of features including seed size, shape, and color.

### 4.4. Statistical Analysis

Principal component analysis (PCA) was carried out on the raw data to simplify and reduce the dimension of the data under investigation. The first 20 principal components with the most variance was used for the linear discriminant analysis (LDA).

Moreover, standardization of all morpho-colorimetric features was executed, and for each statistical comparison, in order to evaluate how well each level of independent variable contributed to the model, the Wilks’ lambda, the percentage of explained variance, the canonical correlation between the discriminant functions, and the group membership were computed. In addition, Box’s M test was executed to assess the homogeneity of the covariance matrices of the best features chosen by the LDA, and in order to verify the homoscedasticity of the variance of the dependent variables, the standardized residual was performed [48]. Finally, in order to compare the empirical distribution of the discriminant functions and the relative cumulative distribution, the Kolmogorov–Smirnov test was performed. Levene’s test was executed to assess the equality of variances for the discriminant functions used [49]. Finally, the data were statistically elaborated by the stepwise LDA method to compare and discriminate each accession and taxon. LDA is commonly used to classify and identify unknown groups using quantitative and qualitative variables [50]. LDA was able to select the different predictor variables entered into the database, minimizing the within-class distances and maximizing the between-class distances, thus, achieving maximum discrimination [51,52,53,54]. The stepwise method, using three statistical variables, tolerance, F-to-enter, and F-to-remove, identifies and selects the best features and uses them to identify seed samples. The tolerance value indicates the proportion of the variance of a variable that is not accounted for by other independent variables in the equation. The F-to-enter and F-to-remove values define the power of each variable in the model and describe what happens if a variable is either inserted or removed from the current model. At each step, the variable with the largest F-to-enter value exceeding the selected entry criteria (F ≥ 3.84) is added to the model, while those with lower values are excluded. The process is automatically stopped when the highest discrimination is reached [55]. Finally, a cross-validation procedure is applied to verify the performance of the discrimination system. PCA was performed using the software R 3.6.3 (R Core Team, 2020, Vienna, Austria). LDAs were executed using the IBM SPSS 16.0 software (Statistical Package for Social Science, IBM Corp., Armonk, NY, USA). 

## 5. Conclusions

This study underlines ecology as the main factor influencing the observed similarities and dissimilarities, because the separation between the mountain and the coastal taxa is high. However, the separation among taxa within the coastal and the mountain groups, and the separation among populations within taxa, were much lower than the separation among the mountain and the coastal groups. Therefore, we suppose that a complex network of gene flow among taxa within groups and among populations within taxa is still active, probably maintained by rare but possible long-distance dispersal of seeds due to seed flotation on the sea surface and by the pollination network. In agreement with previous studies, our data suggest that not only past evolutionary events, but also pollinator movements and seed dispersal patterns, can be major drivers of the evolutionary trajectories of the studied species.

## Figures and Tables

**Table 1:** Percentages of correct classification among coastal and mountainous *Anchusa* species endemic to Sardinia. In bold, the correct classification; in parentheses, the number of analyzed seeds.

Ecological Group	Coastal Taxa	Mountainous Taxa	Total
Coastal taxa	**90.8** (1185)	9.2 (120)	100 (1305)
Mountainous taxa	12.6 (175)	**87.4** (1212)	100 (1387)
Cross-validated			89.0% (2692)

**Table 2:** Percentages of correct classification of the Sardinian *Anchusa* species. In bold, the correct classification; in parentheses, the number of analyzed seeds.

Ecological Conditions	Coastal Taxa				Mountainous Taxa			
Taxa	*A. crispa* ssp. *crispa*	*A. crispa* ssp. *maritima*	*A. littorea*	*A. sardoa*	*A. capellii*	*A. formosa*	*A. montelinasana*	Total
*A. crispa* ssp. *crispa*	**70.7** (191)	14.1 (38)	1.1 (3)	8.1 (22)	5.2 (14)	0.4 (1)	0.4 (1)	100 (270)
*A. crispa* ssp. *maritima*	21.1 (111)	**34.9** (183)	10.3 (54)	19.0 (100)	9.5 (50)	1.7 (9)	3.4 (18)	100 (525)
*A. littorea*	3.5 (14)	5.5 (22)	**81.2** (324)	4.0 (16)	-	2.8 (11)	3.0 (12)	100 (399)
*A. sardoa*	3.6 (4)	7.2 (8)	-	**82.9** (92)	4.5 (5)	1.8 (2)	-	100 (111)
*A. capellii*	4.5 (13)	8.0 (23)	2.4 (7)	4.2 (12)	**71.3** (204)	4.2 (12)	5.2 (15)	100 (286)
*A. formosa*	2.2 (17)	3.6 (28)	2.4 (19)	2.3 (18)	9.4 (74)	**53.9** (424)	26.2 (206)	100 (786)
*A. montelinasana*	2.5 (8)	3.2 (10)	0.3 (1)	2.5 (8)	8.9 (28)	26.0 (82)	**56.5** (178)	100 (315)
Cross-validated (%)								59.3% (2692)

**Table 3:** List of the 74 morpho-colorimetric parameters measured on each seed variant and calculated by Particles8 plugins from ImageJ v. 1.49.

Morphometric Parameters	Description	Colorimetric Parameters	Description
*Perim*	Perimeter, calculated from the centres of the boundary pixels	*GrIntDen*	Greyscale integrated density (the sum of the greyscale values in the particle)
*Area*	Area inside the polygon defined by the perimeter	*GrMin*	Minimum greyscale
*Pixels*	Number of pixels forming the endocarp image	*GrMax*	Maximum greyscale
*MinR*	Radius of the inscribed circle centred at the middle of mass	*GrMode*	Modal greyscale
*MaxR*	Radius of the enclosing circle centred at the middle of mass	*GrMedian*	Median greyscale
*Feret*	Largest axis length	*GrAverage*	Average greyscale
*Breadth*	Largest axis perpendicular to the Feret	*GrAvDeve*	Average deviation of greyscale
*CHull*	Convex hull or convex polygon calculated from pixel centres	*GrStDev*	Standard deviation of the greyscale
*CArea*	Area of the convex hull polygon	*GrVa*	Variance of the greyscale values
*MBCRadius*	Radius of the minimal bounding circle	*GrSkew*	Skewness of the greyscale
*AspRatio*	Aspect ratio = Feret/Breadth	*GrKurt*	Kurtosis of the greyscale
*Circ*	Circularity = 4·π·Area/Perimeter^2^	*GrEntr*	Entropy of the greyscale
*Roundness*	Roundness = 4·Area/(π·Feret^2^)	*RedIntDen*	Redscale integrated density
*ArEquivD*	Area equivalent diameter = √((4/π)·Area)	*RedMin*	Minimum redscale
*PerEquivD*	Perimeter equivalent diameter = Area/π	*RedMax*	Maximum redscale
*EquivEllAr*	Equivalent ellipse area = (π·Feret·Breadth)/4	*RedMode*	Modal redscale
*Compactness*	Compactness = √((4/π)·Area)/Feret	*RedMedian*	Median redscale
*Solidity*	Solidity = Area/Convex_Area	*RedAverage*	Average redscale
*Concavity*	Concavity = Convex_Area-Area	*RedAvDev*	Average deviation of redscale
*Convexity*	Convexity = Convex_hull/Perimeter	*RedStDev*	Standard deviation of the redscale
*Shape*	Shape = Perimeter^2^/Area	*RedVar*	Variance of the redscale
*RFactor*	RFactor = Convex_Hull /(Feret·π)	*RedSkew*	Skewness of the redscale
*ModRatio*	Modification ratio = (2·MinR)/Feret	*RedKurt*	Kurtosis of the redscale
*Sphericity*	Sphericity = MinR/MaxR	*RedEntr*	Entropy of the redscale
*ArBBox*	Area of the bounding box along the feret diameter = Feret·Breadth	*GreenIntDen*	Greenscale integrated density
*Rectang*	Rectangularity = Area/ArBBox	*GreenMin*	Minimum greenscale
		*GreenMax*	Maximum greenscale
		*GreenMode*	Modal greenscale
		*GreenMedian*	Median greenscale
		*GreenAverage*	Average greenscale
		*GreenAvDev*	Average deviation of greenscale
		*GreenStDev*	Standard deviation of the greenscale
		*GreenVar*	Variance of the greenscale
		*GreenSkew*	Skewness of the greenscale
		*GreenKurt*	Kurtosis of the greenscale
		*GreenEntr*	Entropy of the greenscale
		*BlueIntDen*	Bluescale integrated density
		*BlueMin*	Minimum bluescale
		*BlueMax*	Maximum bluescale
		*BlueMode*	Modal bluescale
		*BlueMedian*	Median bluescale
		*BlueAverage*	Average bluescale
		*BlueAvDev*	Average deviation of bluescale
		*BlueStDev*	Standard deviation of the bluescale
		*BlueVar*	Variance of the bluescale
		*BlueSkew*	Skewness of the bluescale
		*BlueKurt*	Kurtosis of the bluescale
		*BlueEntr*	Entropy of the bluescale

## Supplementary Materials

The following are available online at https://www.mdpi.com/2223-7747/9/10/1321/s1, Table S1: Importance of principal components, Table S2: Percentage of correct classification of the 17 *Anchusa* populations analyzed. In bold, the correct classification; in parentheses, the number of analyzed seeds, Table S3: Accessions of *Anchusa* taxa with the collecting localities and number of seeds analyzed in this study. Accession numbers refer to the Sardinian Germplasm Bank (BG-SAR) of Hortus Botanicus Karalitanus of the University of Cagliari, Figure S1: Percentage of explained variance of the first 10 principal components, Figure S2: Contribution of variables to PC1. Figure S3: Contribution of variables to PC2.
