# Supplementary material for: Morpho-Colorimetric Characterization of the Sardinian Endemic Taxa of the Genus Anchusa L. by Seed Image Analysis
Source: Plants (Basel). 2020 Oct 6;9(10):1321. doi: 10.3390/plants9101321 (PMC7601759; doi:10.3390/plants9101321)
Supplement: Supplementary file 1 [file plants-09-01321-s001.pdf]

Supplementary materials

Morpho-colorimetric characterisation of the Sardinian endemic taxa of the genus *Anchusa* L. by seed image analysis

Emmanuele Farris [1], Martino Orrù [2], Mariano Ucchesu [3], Arianna Amadori [1], Marco Porceddu [3-4*] and Gianluigi Bacchetta [3-4]

[1] Dipartimento di Chimica e Farmacia, Università di Sassari, Via Piandanna 4, 07100, Sassari, Italy; emfa@uniss.it (E.F.); ariannaamadori81@gmail.com (A.M.)
[2] Independent researcher, via Nazionale 09023 Monastir (CA) Italy; martino.orru@gmail.com (M.O.)
[3] Centre for the Conservation of Biodiversity (CCB), Life and Environmental Sciences Department, University of Cagliari (DiSVA), Viale S. Ignazio da Laconi 11-13, 09123 Cagliari, Italy; marianoucchesu@gmail.com (M.U.); porceddu.marco@unica.it (M.P.); bacchet@unica.it (G.B.)
[4] Sardinian Germplasm Bank (BG-SAR), Hortus Botanicus Karalitanus (HBK), University of Cagliari, Viale S. Ignazio da Laconi, 9-11, Cagliari 09123, Italy; (M.P. and G.B.)
* Correspondence: porceddu.marco@unica.it (M.P.)

Table S1. Importance of principal components.

	Comp.1	Comp.2	Comp.3	Comp.4	Comp.5	Comp.6	Comp.7	Comp.8
Standard deviation	4.8017617	3.7709981	3.6590353	2.47533805	1.83921035	1.75996246	1.57552096	1.2129034
Proportion of Variance	0.3115799	0.1921679	0.1809262	0.08280133	0.04571209	0.04185767	0.03354414	0.0198802
Cumulative Proportion	0.3115799	0.5037479	0.6846741	0.76747540	0.81318749	0.85504516	0.88858930	0.9084695

	Comp.9	Comp.10	Comp.11	Comp.12	Comp.13	Comp.14	Comp.15
Standard deviation	1.04838379	0.99468223	0.856999251	0.81137211	0.788045875	0.582438090	0.566031221
Proportion of Variance	0.01485282	0.01337017	0.009924969	0.00889628	0.008392112	0.004584245	0.004329613
Cumulative Proportion	0.92332232	0.93669249	0.946617459	0.95551374	0.963905851	0.968490096	0.972819709

	Comp.16	Comp.17	Comp.18	Comp.19	Comp.20	Comp.21	Comp.22
Standard deviation	0.518946513	0.475544336	0.441617121	0.430929012	0.402695207	0.348176110	0.317819460
Proportion of Variance	0.003639263	0.003055979	0.002635482	0.002509457	0.002191398	0.001638197	0.001364989
Cumulative Proportion	0.976458972	0.979514951	0.982150433	0.984659890	0.986851288	0.988489485	0.989854474

	Comp.23	Comp.24	Comp.25	Comp.26	Comp.27	Comp.28
Standard deviation	0.309706945	0.298040534	0.274734963	0.2660486342	0.2191045116	0.2140639175
Proportion of Variance	0.001296194	0.001200381	0.001019991	0.0009565118	0.0006487404	0.0006192346
Cumulative Proportion	0.991150669	0.992351049	0.993371040	0.9943275518	0.9949762921	0.9955955268

	Comp.29	Comp.30	Comp.31	Comp.32	Comp.33	Comp.34
Standard deviation	0.2076507705	0.1954182017	0.1644637315	0.1582033478	0.1509613149	0.1472839436
Proportion of Variance	0.0005826871	0.0005160578	0.0003655178	0.0003382203	0.0003079638	0.0002931427
Cumulative Proportion	0.9961782138	0.9966942716	0.9970597894	0.9973980096	0.9977059734	0.9979991161

	Comp.35	Comp.36	Comp.37	Comp.38	Comp.39	Comp.40
Standard deviation	0.1377374854	0.1223172768	0.1141197474	0.1062568363	0.1052324133	0.1030232295
Proportion of Variance	0.0002563732	0.0002021827	0.0001759908	0.0001525745	0.0001496468	0.0001434295
Cumulative Proportion	0.9982554893	0.9984576719	0.9986336627	0.9987862372	0.9989358840	0.9990793135

	Comp.41	Comp.42	Comp.43	Comp.44	Comp.45	Comp.46
Standard deviation	0.0958766887	0.0919886599	8.363156e-02	7.433627e-02	7.186546e-02	7.125183e-02
Proportion of Variance	0.0001242208	0.0001143502	9.451673e-05	7.467408e-05	6.979248e-05	6.860572e-05
Cumulative Proportion	0.9992035343	0.9993178845	9.994124e-01	9.994871e-01	9.995569e-01	9.996255e-01

	Comp.47	Comp.48	Comp.49	Comp.50	Comp.51	Comp.52
Standard deviation	6.135126e-02	5.846623e-02	5.421365e-02	0.0529820868	4.918532e-02	4.878554e-02
Proportion of Variance	5.086456e-05	4.619325e-05	3.971783e-05	0.0000379338	3.269184e-05	3.216255e-05
Cumulative Proportion	9.996763e-01	9.997225e-01	9.997622e-01	0.9998001830	9.998329e-01	9.998650e-01

	Comp.53	Comp.54	Comp.55	Comp.56	Comp.57	Comp.58
Standard deviation	4.183564e-02	3.983289e-02	3.817501e-02	3.446232e-02	3.057972e-02	2.630833e-02
Proportion of Variance	2.365163e-05	2.144134e-05	1.969366e-05	1.604935e-05	1.263674e-05	9.353087e-06
Cumulative Proportion	9.998887e-01	9.999101e-01	9.999298e-01	9.999459e-01	9.999585e-01	9.999679e-01

	Comp.59	Comp.60	Comp.61	Comp.62	Comp.63	Comp.64
Standard deviation	2.093096e-02	1.995114e-02	1.934485e-02	1.739908e-02	1.703772e-02	1.350718e-02
Proportion of Variance	5.920337e-06	5.379027e-06	5.057071e-06	4.090919e-06	3.922757e-06	2.465458e-06
Cumulative Proportion	9.999738e-01	9.999792e-01	9.999842e-01	9.999883e-01	9.999922e-01	9.999947e-01

	Comp.65	Comp.66	Comp.67	Comp.68	Comp.69	Comp.70
Standard deviation	1.133304e-02	1.037604e-02	9.097449e-03	8.156933e-03	2.617155e-03	2.055661e-04
Proportion of Variance	1.735645e-06	1.454894e-06	1.118427e-06	8.991290e-07	9.256080e-08	5.710462e-10
Cumulative Proportion	9.999964e-01	9.999979e-01	9.999990e-01	9.999999e-01	1.000000e+00	1.000000e+00

	Comp.71	Comp.72	Comp.73	Comp.74
Standard deviation	4.816940e-05	1.243993e-05	2.725803e-06	6.106621e-08
Proportion of Variance	3.135528e-11	2.091241e-12	1.004054e-13	5.039300e-17
Cumulative Proportion	1.000000e+00	1.000000e+00	1.000000e+00	1.000000e+00

Table S2. Percentage of correct classification of the 17 *Anchusa* populations analysed. In bold, the correct classification; in parentheses, the number of analysed seeds.

**Ecological Conditions**	**Coastal Taxa**													**Mountainous Taxa**				
Taxon	*A. crispa* ssp. *crispa*						*A. crispa* ssp. *maritima*			*A. littorea*			*A. sardoa*	*A. capellii*	*A. formosa*		*A. montelinasana*	
Populations	FS	POR	SP	EM	PP	AS	FC	SPM	TV	IA	SAN	SAR	PC	MS	ML	SS	GON	Total
FS	**39.6** (19)	4.2 (2)	4.2 (2)	12.5 (6)	8.3 (4)	8.3 (4)	6.3 (3)	6.3 (3)	-	-	-	-	10.4 (1)	-	-	-	-	100 (48)
POR	2.2 (1)	**73.3** (33)	-	4.4 (2)	6.7 (3)	6.7 (3)	-	-	-	-	-	-	6.7 (3)	-	-	-	-	100 (45)
SP	12.3 (7)	3.5 (2)	**24.6** (14)	19.3 (11)	-	12.3 (7)	10.5 (6)	3.5 (2)	5.3 (3)	1.8 (1)	-	-	5.3 (3)	-	1.8 (1)	-	-	100 (57)
EM	8.7 (2)	-	8.7 (2)	**60.9** (14)	-	17.4 (4)	4.3 (1)	-	-	-	-	-	-	-	-	-	-	100 (23)
PP	11.1 (4)	2.8 (1)	2.8 (1)	5.6 (2)	**52.8** (19)	11.1 (4)	8.3 (3)	-	-	-	-	-	5.6 (2)	-	-	-	-	100 (36)
AS	4.9 (3)	16.4 (10)	4.9 (3)	9.8 (6)	1.6 (1)	**41.0** (25)	1.6 (1)	4.9 (3)	9.8 (6)	-	-	-	-	3.3 (2)	1.6 (1)	-	-	100 (61)
FC	2.7 (8)	3.4 (10)	4.4 (13)	2.7 (8)	5.5 (16)	1.7 (5)	**32.8** (96)	2.4 (7)	2.7 (8)	14.3 (42)	1.7 (5)	1.0 (3)	16.7 (49)	2.0 (6)	2.4 (7)	2.0 (6)	1.4 (4)	100 (293)
SPM	7.8 (11)	4.3 (6)	6.4 (9)	7.1 (10)	1.4 (2)	2.1 (3)	5.0 (7)	**43.3** (61)	8.5 (12)	1.4 (2)	-	-	10.6 (15)	2.1 (3)	-	-	-	100 (141)
TV	2.2 (2)	2.2 (2)	2.2 (2)	2.2 (2)	2.2 (2)	5.5 (5)	6.6 (6)	2.2 (2)	**46.2** (42)	-	-	-	6.6 (6)	22.0 (20)	-	-	-	100 (91)
IA	0.5 (1)	0.5 (1)	-	1.4 (3)	5.2 (11)	0.5 (1)	12.7 (27)	0.5 (1)	-	**50.9** (108)	17.0 (36)	5.2 (11)	1.9 (4)	-	1.4 (3)	1.4 (3)	0.9 (2)	100 (212)
SAN	-	-	3.0 (1)	-	-	-	3.0 (1)	-	-	21.2 (7)	**69.7** (23)	3.0 (1)	-	-	-	-	-	100 (33)
SAR	0.6 (1)	-	0.6 (1)	-	0.6 (1)	-	3.9 (6)	-	-	5.8 (9)	26.6 (41)	**61.7** (95)	-	-	-	-	-	100 (154)
PC	-	5.4 (6)	-	-	5.4 (6)	0.9 (1)	3.6 (4)	4.5 (5)	0.9 (1)	-	-	-	**74.8** (83)	1.8 (2)	0.9 (1)	-	1.8 (2)	100 (111)
MS	1.0 (3)	7.7 (22)	-	-	1.7 (5)	1.7 (5)	3.5 (10)	1.7 (5)	12.9 (37)	2.4 (7)	0.7 (2)	0.3 (1)	3.1 (9)	**52.4** (150)	2.8 (8)	3.5 (10)	4.2 (12)	100 (286)
ML	1.3 (8)	2.2 (14)	-	-	1.0 (6)	0.8 (5)	4.0 (25)	0.8 (5)	2.2 (14)	1.4 (9)	0.6 (4)	-	1.7 (11)	7.5 (47)	**39.0** (246)	20.5 (129)	17.0 (107)	100 (630)
SS	3.2 (5)	0.6 (1)	-	-	1.9 (3)	-	1.3 (2)	-	0.6 (1)	1.9 (3)	-	-	-	0.6 (1)	11.5 (18)	**69.2** (108)	9.0 (14)	100 (156)
GON	2.5 (8)	0.3 (1)	0.6 (2)	0.3 (1)	2.5 (8)	0.6 (2)	1.6 (5)	0.3 (1)	0.6 (2)	1.9 (6)	-	-	1.0(3)	7.0 (22)	18.4 (58)	21.6 (68)	**40.6** (128)	100 (315)
Cross-validated (%)																		47.0% (2,692)

Table S3. Accessions of *Anchusa* taxa with the collecting localities and number of seeds analysed in this study. Accession numbers refer to the Sardinian Germplasm Bank (BG-SAR) of Hortus Botanicus Karalitanus of the University of Cagliari.

Taxon	Population Code	Accession Number	Coordinates	Locality	Number of Seeds
Anchusa capellii	**MS**	297/05	39°44'57.47" N 9°17'58.00" E	Monte Santa Vittoria (Esterzili, CA)	95
Anchusa capellii		441/04		Monte Santa Vittoria (Esterzili, CA)	191
Anchusa crispa ssp. *crispa*	**FS**	79/16	40°50'37.94" N 8°18'56.71" E	Fiume Santo (Porto Torres, SS)	48
Anchusa crispa ssp. *crispa*	**POR**	80/16	40°38'35.97" N 8°11'19.74" E	Porticciolo (Alghero, SS)	45
Anchusa crispa ssp. *crispa*	**SP**	81/16	40°51'47.39" N 8°16'55.30" E	Stagno di Pilo (Sassari, SS)	57
Anchusa crispa ssp. *crispa*	**EM**	82/16	40°52'46.25" N 8°15'44.54" E	Ezzi Mannu (Sassari, SS)	23
Anchusa crispa ssp. *crispa*	**PP**	83/16	40°44'55.36" N 8° 9'29.40" E	Porto Palmas (Argentiera, SS)	36
Anchusa crispa ssp. *crispa*	**AS**	86/16	40°59'20.39" N 8°13'20.68" E	Asinara (Porto Torres, SS)	61
Anchusa crispa ssp. *maritima*	**FC**	198/06	40°56'22.89" N 8°49'1.37" E	Foce del Coghinas (Badesi, SS)	77
Anchusa crispa ssp. *maritima*		208/12		Foce Coghinas (Badesi, SS)	116
Anchusa crispa ssp. *maritima*		85/16		Foci del Coghinas (Badesi, SS)	100
Anchusa crispa ssp. *maritima*	**SPM**	84/16	40°55'34.58" N 8°47'50.94" E	San Pietro a Mare (Valledoria, SS)	141
Anchusa crispa ssp. *maritima*	**TV**	87/16	41° 7'36.10" N 9° 3'38.97" E	Torre di Vignola (Trinità d'Agultu, SS)	91
Anchusa formosa	**ML**	496/04	39° 8'58.67" N 8°50'29.71" E	Monte Lattias (Uta, CA)	201
Anchusa formosa		188/05°		Monte Lattias (Uta, CA)	95

Anchusa formosa		303/05		Monte Lattias (Uta, CA)	102
Anchusa formosa		357/05		Monte Lattias (Uta, CA)	21
Anchusa formosa		331/12		Monte Lattias (Uta, CA)	211
Anchusa formosa	**SS**	301/05	39°11'18.14" N 8°51'8.08" E	Su Scavoni (Uta, CA)	102
Anchusa formosa		559/04		Su Scavoni (Uta, CA)	54
Anchusa littorea	**IA**	70/06	39°31'8.91" N 8°25'58.76" E	Is Arenas (Arbus, SU)	73
Anchusa littorea		49/07		Is Arenas (Arbus, SU)	65
Anchusa littorea		104/16		Is Arenas (Arbus, SU)	30
Anchusa littorea		105/16		Is Arenas (Arbus, SU)	20
Anchusa littorea		158/16		Is Arenas (Arbus, SU)	24
Anchusa littorea	**SAN**	106/16	39° 3'45.07" N 8°30'45.06" E	Le saline (Sant'Antioco, SU)	10
Anchusa littorea		157/16		Le saline (Sant'Antioco, SU)	23
Anchusa littorea	**SAR**	107/16	39°49'35.51" N 8°33'9.94" E	Stagno S'Ena Rubia (Arborea, OR)	20
Anchusa littorea		155/16		Stagno S'Ena Rubia (Arborea, OR)	100
Anchusa littorea		156/16		Stagno S'Ena Rubia (Arborea, OR)	14
Anchusa littorea		159/16		Stagno S'Ena Rubia (Arborea, OR)	20
Anchusa montelinasana	**GON**	CP 01/05	39°27'0.00" N 8°37'0.00" E	Monte Linas (Gonnosfanadiga, SU)	145
Anchusa montelinasana		CP 11/06		Monte Linas (Gonnosfanadiga, SU)	170
Anchusa sardoa	**PC**	78/16	40°37'4.91" N 8°12'4.98" E	Mugoni (Alghero, SS)	111

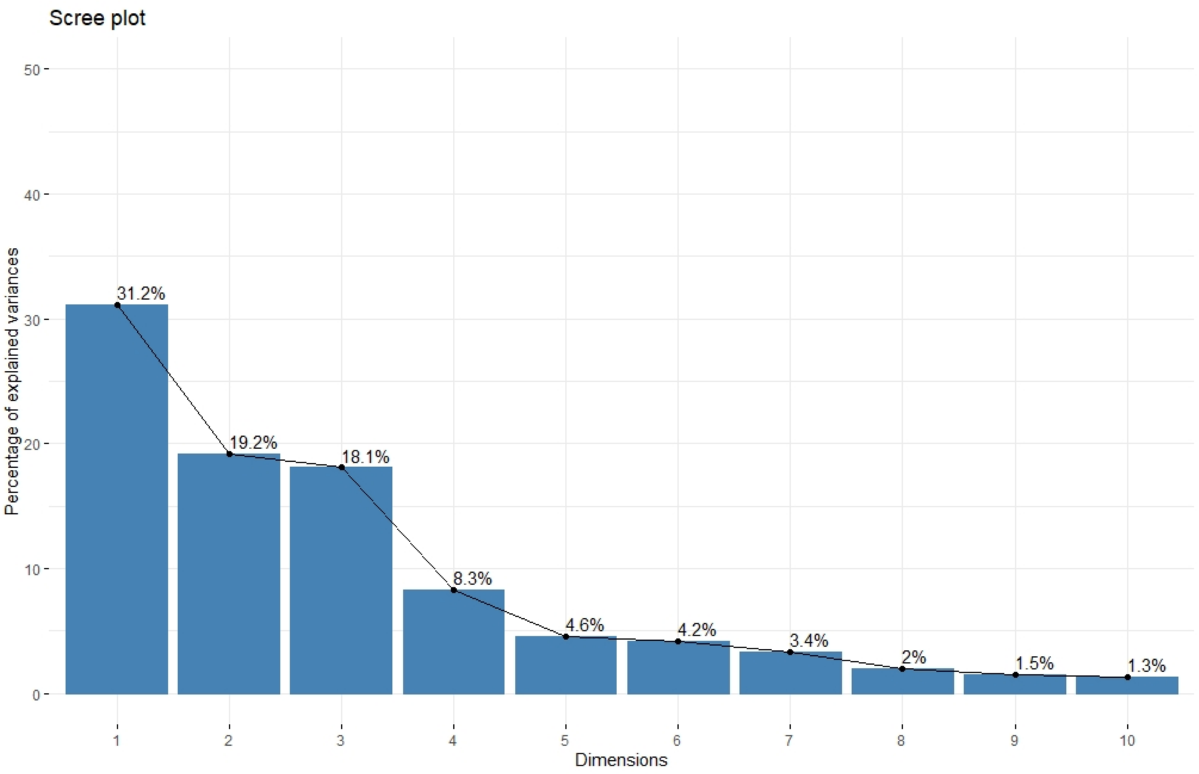

Figure S1. Percentage of explained variance of the first 10 principal component.

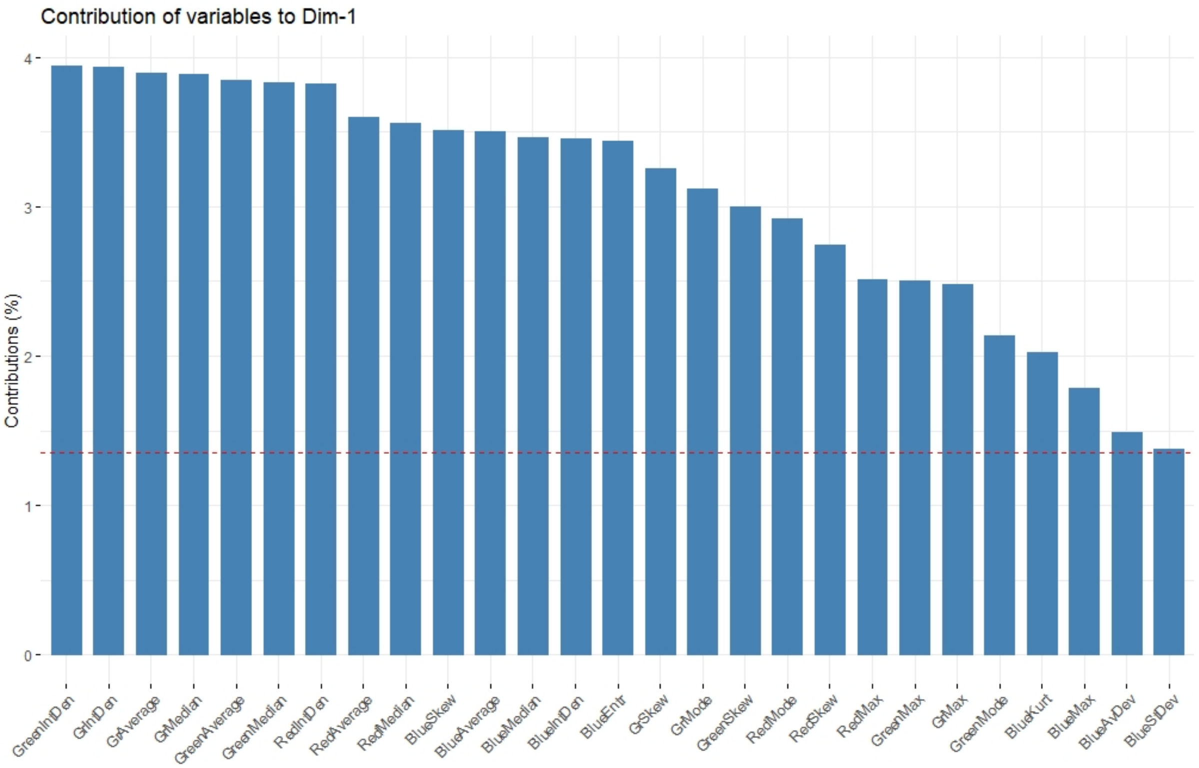

Figure S2. Contribution of variables to PC1.

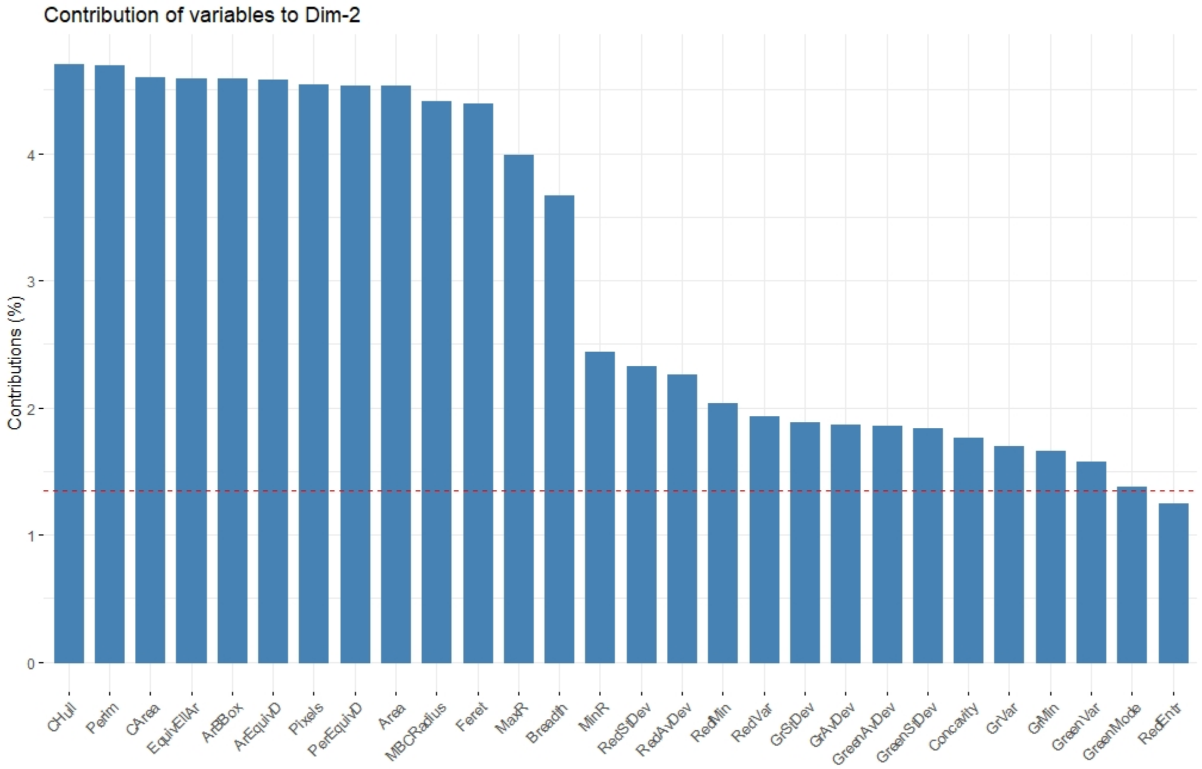

Figure S3. Contribution of variables to PC2.
